# Evaluation of the Concentration of Ethanol as a Vehicle for the Administration of Resveratrol Measured Through Its Antioxidant Effect in the Hippocampus of Wistar Rats

**DOI:** 10.1155/jnme/6614635

**Published:** 2025-09-30

**Authors:** Addí Rhode Navarro-Cruz, Ivan Cesar-Arteaga, Daniel Juárez-Serrano, Raúl Ávila-Sosa Sánchez, Teresa Soledad Cid-Pérez, Obdulia Vera-López, Gladys Quezada-Figueroa, Ashuin Kammar-García, Orietta Segura-Badilla

**Affiliations:** ^1^Departamento de Bioquímica-Alimentos, Facultad de Ciencias Químicas, Benemérita Universidad Autónoma de Puebla, Puebla, Mexico; ^2^Departamento de Nutrición y Salud Pública, Facultad de Ciencias de la Salud y los Alimentos, Universidad del Bío-Bío, Bío-Bío, Concepción, Chile; ^3^Dirección de Investigación, Instituto Nacional de Geriatría, Mexico City, Mexico; ^4^Department of Global Health and Population, Harvard TH Chan School of Public Health, Boston, Massachusetts, USA

**Keywords:** antioxidants, drug vehicles, ethanol, oxidative stress, resveratrol

## Abstract

**Introduction:** Oxidative stress is an imbalance between endogenous antioxidants and oxidizing molecules, the latter having an unpaired electron in their last valence layer, with those derived from oxygen and nitrogen being the most important. Resveratrol is a natural polyphenol with antioxidant properties that reduce oxidative stress. However, the bioavailability of resveratrol is low due to its rapid excretion and extensive metabolism. Polyphenols improve their bioavailability in the presence of ethanol, a popular substance present in alcoholic beverages, which is used as a vehicle in the pharmaceutical industry. The objective of this work was to determine the optimal concentration of ethanol for the administration of resveratrol at a concentration of 10 mg/kg/day. Fifty-four Wistar male rats were randomly allocated to nine groups: control (water), four vehicle groups (2.5%, 5%, 7.5%, and 10% ethanol v/v), and four resveratrol + vehicle groups (10 mg/kg/day + ethanol concentrations 2.5%–10%) (ResVitále®, *Polygonum cuspidatum*, USA). Posterior to administration, the hippocampus of Wistar rats was analyzed for oxidative stress and enzyme activity. Nitric oxide levels and lipid peroxidation products were significantly lower with 7.5% ethanol, compared to 10% ethanol. Resveratrol modulated CAT and SOD activity at certain ethanol concentrations, with a limited overall effect. In conclusion, 7.5% ethanol is the most optimal vehicle concentration for enhancing the antioxidant effects of resveratrol.

## 1. Introduction

Oxidative stress is generated by an imbalance in the endogenous oxidant–antioxidant system, decreasing antioxidant capacity and increasing free radicals and oxidative effects [1]. Macromolecules and complex cellular structures formed by proteins, lipids, and DNA are damaged under oxidative stress. The main free radicals involved are superoxide anion (•O_2_), nitric oxide (•NO), hydroxyl radical (•OH), peroxyl radical (ROO), peroxynitrite (ONOO-), and hypochlorous acid (HOCl). The latter are not necessarily free radicals but are highly toxic to the cell and promote the generation of free radicals. When this imbalance occurs, damage to macromolecules compromises the structural and functional integrity of the cell [2].

Living beings possess an important network of endogenous antioxidant defenses, which eliminate, reduce, or repair damage caused by free radicals and reactive species [3]. When endogenous antioxidants are insufficient, it becomes necessary to use alternative compounds, such as vitamin E, curcumin, and resveratrol, that help counteract the structural and functional damage that can affect various organs and systems.

The hippocampus is particularly vulnerable to oxidative stress due to its high metabolic activity, relatively low antioxidant defenses, and central role in learning and memory processes [4]. This region integrates and relays information from cortical areas through circuits such as the entorhinal-hippocampal loop and the Papez circuit, and its plasticity, partially maintained by ongoing neurogenesis in the dentate gyrus, makes it highly sensitive to external insults, including substance exposure during early life stages [5–9].

Resveratrol has gained importance in recent decades due to its anticarcinogen, antiaging, and antioxidant properties [10]. One of the most common ways to administer resveratrol is the oral route. However, the poor solubility of this molecule in water represents a challenge [11].

Liquid dosage forms are generally used to administer exogenous antioxidants orally, through mixtures made up of the active ingredient, excipients, and a vehicle. The vehicle is generally a liquid substance used to dissolve the active substance, which can be polar, semipolar or nonpolar. Sterile distilled water (aqueous vehicle), vegetable oils (oily vehicle), or ethanol (alcoholic vehicle) are commonly used. When alcohol is used, declaring the alcoholic content on the label is required due to the risk it may impose to patients with liver disease, alcoholism, epilepsy, pregnant women, and children [12].

Chronic alcohol intake is associated with risk of cancer, kidney damage, heart failure, cirrhosis, and dementia [13–15]. However, the use of ethanol as a vehicle allows the absorption of polyphenols, increasing their bioavailability [16, 17]. Previous in vitro research has demonstrated that ethanol can enhance the solubility, stability, and cellular uptake of resveratrol, potentially improving its antioxidant and therapeutic effects [18]. However, these studies did not explore the comparative efficacy or safety of using ethanol at varying concentrations as a vehicle. The hurdle of effectively delivering resveratrol has prompted extensive investigation into various strategies, including its structural modification. Moreover, the limited availability of nontoxic excipients capable of enhancing resveratrol solubility remains a significant challenge. The optimal dose of ethanol required to effectively deliver resveratrol at a concentration of 10 mg/kg/day is not known. Results from our laboratory have shown that a prolonged administration of ethanol at low doses increases oxidative stress in the hippocampus of rats [19, 20], and recently, we published evidence showing that resveratrol co-administered with ethanol at concentrations ranging from 10% to 50% reduced oxidative stress markers in the hippocampus of Wistar rats exposed to chronic ethanol intake [13]. Nonetheless, in that study, the ethanol concentration was used to model different types of alcoholic beverages, and the role of ethanol as a delivery vehicle was not the primary focus. Given the widespread interest in resveratrol as a neuroprotective compound and the potential translational relevance of optimizing its delivery, identifying the most effective and least harmful ethanol concentration may inform future strategies for therapeutic formulations. We hypothesize that ethanol, when used at low concentrations (2.5%–10%), can act as an effective vehicle to enhance the antioxidant effects of resveratrol in the hippocampus, without inducing toxicity. Therefore, the present study aims to address this gap by evaluating the antioxidant effects of resveratrol administered with ethanol at low concentrations (2.5%–10%) in order to determine the optimal ethanol concentration to effectively serve as a vehicle for resveratrol administration at 10 mg/kg/day.

This in vivo approach contrasts with previous studies, which have focused mainly on in vitro solubility or used high ethanol concentrations to model alcoholic beverages.

## 2. Materials and Methods

### 2.1. Experimental Design

Fifty-four male Wistar rats (5 months old, weighing 280–320 g at the start of the experiment) were obtained from the Claude Bernard Biotery of the Benemérita Universidad Autónoma de Puebla. The animals were kept in standard vivarium conditions with a 12:12 h light-dark cycle and a constant temperature of 21 ± 1°C. Food and water were provided ad libitum.

All procedures were approved by the Institutional Committee for the Care and Use of Laboratory Animals and carried out in accordance with the Official Mexican Standard NOM-62-ZOO-1999, as well as the Mexican “Guide for the Care and Use of Laboratory Animals” [21]. All procedures were carried out minimizing animal suffering.

#### 2.1.1. Resveratrol Administration

The animals were randomly allocated to nine groups: control (water), four vehicle groups (2.5%, 5%, 7.5%, and 10% ethanol v/v), and four resveratrol + vehicle groups (10 mg/kg/day + ethanol concentrations 2.5%–10%) (ResVitále®, *Polygonum cuspidatum*, USA).

Ethanol solutions (density *ρ* = 0.789 g/mL) were formulated by diluting with distilled water to achieve concentrations of 2.5%, 5%, 7.5%, and 10%. These concentrations correspond to doses of 19.7, 39.4, 59.1, and 78.9 mg/kg, respectively. Oral administration was performed for all groups through an intragastric tube (16 gauge with a ball-shaped tip).

For the administration of resveratrol, the solution was prepared in a water–ethanol vehicle at 2.5%, 5%, 7.5%, and 10%, in a concentration of 5 mg/mL. The selection of ethanol concentrations was guided by practical and experimental considerations. These values were intentionally chosen to remain within a low and pharmacologically acceptable range, based on precedents in pharmaceutical formulation and experimental toxicology [12]. Ethanol at concentrations below 10% is widely recognized as a safe solvent for oral administration in animal models, offering sufficient solubilizing capacity for polyphenolic compounds while minimizing systemic and neurological toxicity. Prior studies have reported the use of ethanol in formulations ranging from 5% to 17% in similar contexts [12, 22]. In addition, previous work from our group demonstrated that chronic administration of low-dose ethanol can alter oxidative stress markers in the hippocampus, even in the absence of other active compounds [19, 20]. The resveratrol dose was selected from a previous publication based on positive results [20]. All treatments were applied during the morning (between 8:00 a.m. and 10:00 a.m.) for 2 months.

Following the final administration, rats were anesthetized via intraperitoneal injection of sodium pentobarbital (75 mg/kg body weight) and subjected to transcardial perfusion with 0.1 M phosphate buffer. The hippocampi were then dissected bilaterally and stored at −70°C (Ultrafreezer 716 Thermo Forma, Dreieich, Alemania) for the subsequent determination of oxidative stress markers. The tissues were homogenized (POLYTRON PT 3100) and centrifuged at 18, 000 × *g* at 4°C for 30 min as previously described by Juárez et al. [7]. The supernatant was used for biochemical determinations.

### 2.2. Biochemical Analysis

#### 2.2.1. Total Protein (TP) Quantification

The concentration of TP was quantified according to the method described by Sedmak and Grossberg [23], using bovine serum albumin as the reference standard. For each assay, 2 μL of supernatant was added to 500 μL of the colorimetric reagent (0.06% Coomassie blue, absorbance maximum at 465 nm), and the volume was adjusted to 1 mL with distilled water. The resulting absorbance was read at 620 nm with the Lambda EZ 150 spectrometer (PerkinElmer, USA). Protein concentrations were determined by interpolating sample optical density (OD) values against a BSA standard curve (1–10 μg). All measurements were conducted in parallel.

#### 2.2.2. Quantification of Nitrites

Nitric oxide production was quantified by measuring nitrite ion (NO_2_) concentration in the supernatant using the Griess method [24]. The colorimetric reaction was induced by adding 100 μL of supernatant, 100 μL of Griess reagent, and 800 μL of H_2_O. Subsequently, the reaction product was read in a spectrophotometer at 540 nm. Nitrite concentration was determined by interpolating sample OD values against a sodium nitrite (NaNO_2_) standard curve for each assay.

#### 2.2.3. Determination of Malondialdehyde (MDA) + 4-Hydroxynonenal (4HDA) and MDA

The generation of lipid peroxidation products, specifically MDA and 4-HDA, was evaluated using the method of Erdelmeier et al. [25], employing N-methyl-2-phenyl-indole as the chromogenic reagent (10.3 mM). A reaction mixture containing 100 μL of distilled water, 100 μL of supernatant, and 650 μL of diluted Solution 1 was shaken vigorously, followed by treatment with 150 μL of methanesulfonic acid (MDA + 4-HDA determination) or 35% HCl (MDA determination). The tubes were incubated at 45°C for 60 min (MDA) and at 45°C for 40 min (MDA + 4-HDA). Following incubation and a 5-min cooling period at room temperature, samples were centrifuged at 3000 rpm for 15 min. Subsequently, the absorbance of the reaction product was read at 586 nm. Concentrations of MDA and 4-HDA were determined by interpolating sample absorbance values against a 1,1,3,3- tetramethoxypropane standard curve prepared for each assay.

### 2.3. Enzymatic Analysis

#### 2.3.1. Catalase (CAT) Activity

CAT enzymatic activity was assessed spectrophotometrically following the procedure outlined by Aebi [26]. The reaction was initiated in a quartz cuvette containing 965 μL of PBS (50 mM), pH = 7.4, 330 μL of H_2_O_2_ (30 mM), and 35 μL of supernatant. The reaction was monitored by measuring the decrease in absorbance at 240 nm at 0 and 2 min, with the temperature maintained at 20°C using a water bath (Julabo TW12). CAT activity was calculated by multiplying the change in ΔOD by the molar extinction coefficient, adjusted for the protein content (mg) of the supernatant.

#### 2.3.2. Determination of Superoxide Dismutase (SOD) Activity

SOD activity was determined by assessing its inhibitory effect on the autoxidation of pyrogallol [27]. A blank reaction (uninhibited autoxidation) was performed, and the volumes of pyrogallol and Tris-HCl buffer were adjusted to achieve a ΔOD of 0.020 ± 0.001. Sample dilutions were performed as necessary to obtain reliable measurements. SOD activity was calculated using the following equation:

(1) $$\begin{array}{c} \text{enzymatic activity}=\left[\frac{\text{mean }\Delta \text{OD value of the sample}\times 100}{\text{mean }\Delta \text{OD value of the blank}}-100\right]\times 0.6. \end{array}$$

All values were normalized to 1 mg of TP in the sample.

### 2.4. Statistical Analysis

Data are presented as mean ± standard error (SE). A two-way fixed-effects analysis of variance (ANOVA) was employed to assess the influence of resveratrol and different vehicle concentrations on the measured parameters of oxidative stress and endogenous antioxidant systems. The factors considered in the ANOVA were vehicle concentration and resveratrol treatment. Post hoc pairwise comparisons were performed using Tukey's test to determine differences between groups.

To ensure the validity of the two-way ANOVA, the assumptions of the model were verified. Independence of observations was ensured through appropriate randomization and experimental design. The normality of residuals was evaluated using graphical methods, including Q-Q plots and histograms. Homogeneity of variances was assessed using Levene's test.

A *p* value of < 0.05 was considered statistically significant. All statistical analyses and graphical representations were generated using GraphPad Prism V.9.0.1.

## 3. Results

### 3.1. Effect of Ethanol Vehicle for Resveratrol Delivery on Nitrite Levels

To assess the influence of the ethanol vehicle on resveratrol's antioxidant activity in the hippocampus, nitrite production, an indirect marker of NO, was quantified. Due to the short half-life of NO (approximately 9 s), its rapid oxidation to nitrates and nitrites necessitates the measurement of these more stable metabolites.

**Figure 1:**
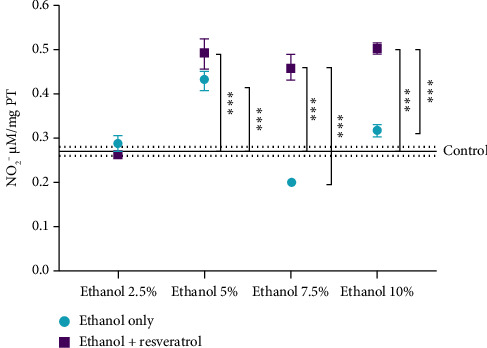
Effect of ethanol as a vehicle for resveratrol on nitrite levels in the hippocampus. Data are presented as mean ± standard error (SE) (*n* = 6 animals per group). The solid line represents the mean nitrite level of the control group, and the dotted line represents the SE. Comparisons were made using a two-way fixed-effects ANOVA model and post hoc comparisons with Tukey's test. ^∗∗∗^*p* < 0.001.

Figure 1 presents the mean nitrate concentrations. The control group exhibited a mean nitrite level of 0.2692 μM/mg protein. At 2.5% ethanol, the difference between the ethanol and resveratrol groups was not significant, nor was the difference compared to the control. However, with 5% ethanol as the vehicle, the vehicle group displayed an 82.50% statistically significant increase in nitrite levels compared to the control, while the resveratrol group showed a 59.73% increase relative to the control; no significant difference was observed between the vehicle and resveratrol groups at this concentration. When 7.5% of ethanol was used as the vehicle, nitrite production increased by 78.41% compared to the control, whereas the resveratrol group showed a significant 24.29% decrease in nitrite levels compared to the control, resulting in a substantial 57.56% reduction compared to the vehicle group. Finally, with 10% ethanol, the vehicle group showed a statistically significant 84.58% increase in nitrite production compared to the control, while the resveratrol group exhibited only a 14.02% increase with respect to the control, representing a decrease of 37.85% compared to the vehicle group.

### 3.2. Effect of Resveratrol on MDA + 4-HDA Production

The main metabolites of lipoperoxidation are MDA and 4-HDA, which are the most studied byproducts of oxidative stress.

**Figure 2:**
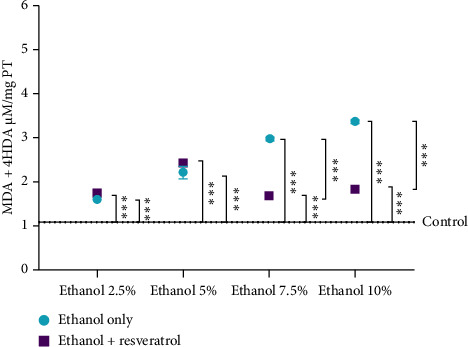
Effect of ethanol on the lipoperoxidation process and the antioxidant activity of resveratrol in the hippocampus. Data are presented as mean ± standard error (SE) (*n* = 6 animals per group). The solid line represents the mean MDA + 4-HDA level of the control group, while the dotted line represents the SE. The SE in this figure may be difficult to visualize due to their low magnitude. Comparisons were made using a two-way fixed-effects ANOVA model and post hoc comparisons with Tukey's test. ^∗∗∗^*p* < 0.001.

Figure 2 shows that administration with 2.5% ethanol resulted in a mean concentration of 1.078 μM/mg protein of MDA + 4-HDA. Compared to the control group, this represents a statistically significant increase of 47.12% in the vehicle group and 59.55% in the resveratrol group. There was no statistically significant difference between the resveratrol and vehicle groups at this concentration. Following administration of 5% ethanol, the vehicle and resveratrol groups exhibited increases of 104.63% and 124.39%, respectively, both statistically significant compared to the control group. Likewise, no statistically significant difference was found between them. At 7.5% ethanol, the vehicle and resveratrol groups showed statistically significant increases of 172.35% and 52.22, respectively. Notably, the administration with resveratrol significantly reduced lipoperoxide production, with a 44.10% decrease compared to the vehicle group. Finally, administration of 10% ethanol led to a 211.41% increase in MDA + 4-HDA levels in the vehicle group compared to the control. In the resveratrol group, a 69.29% increase was observed relative to the control; however, this group showed a statistically significant decrease of 45.63% compared to the vehicle group.

**Figure 3:**
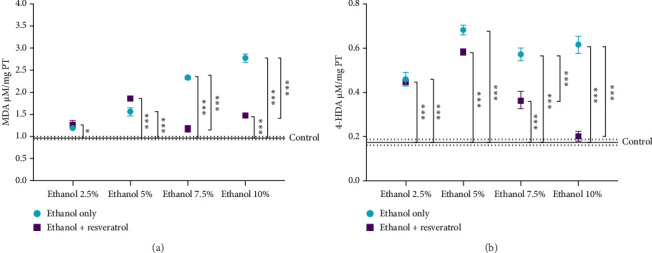
Effect of ethanol on the antioxidant activity of resveratrol as shown by the hippocampal levels of MDA (a) and 4-HDA (b). Data are presented as mean ± standard error (SE) (*n* = 6 animals per group). The solid line represents the mean concentration of the control group, while the dotted line represnts the SE. Comparisons were made using a two-way fixed-effects ANOVA model and post hoc comparisons with Tukey's test. ^∗^*p* < 0.05, ^∗∗^*p* < 0.01, and ^∗∗∗^*p* < 0.001.

MDA is a byproduct of arachidonic acid oxidation, and 4-HDA is produced in smaller quantities. The separate quantification of MDA and 4-HDA is presented in Figure 3.

### 3.3. Evaluation of SOD and CAT Enzymatic Activity by the Effect of Ethanol Vehicle for Resveratrol Delivery

**Figure 4:**
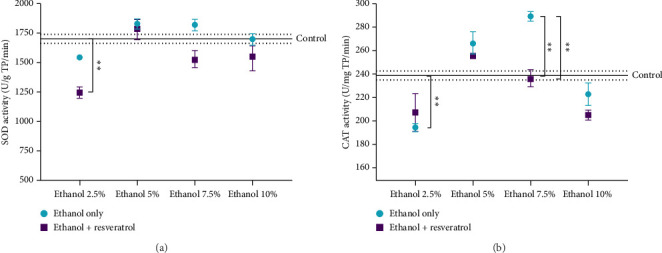
Effect of ethanol as a vehicle for resveratrol on the enzymatic activity of SOD (a) and CAT (b) in the hippocampus. Data are presented as mean ± standard error (SE) (*n* = 6 animals per group). The solid line represents the mean of the control group, and the dotted line represents the SE. Comparisons were made using a two-way fixed-effects ANOVA model and post hoc comparisons with Tukey's test. ^∗^*p* < 0.05 and ^∗∗^*p* < 0.01.

The enzymatic activity of SOD and CAT was assessed 2 months after administration of ethanol at concentrations of 2.5%, 5%, 7.5%, and 10%, either as a vehicle alone or in combination with resveratrol. Significant differences between the ethanol-only and ethanol + resveratrol groups were observed at certain concentrations. Specifically, resveratrol significantly increased SOD activity at 2.5% ethanol, and CAT activity at both 2.5% and 7.5% ethanol. However, at other concentrations, the differences between groups were not statistically significant. The difference between the ethanol and resveratrol groups was not significant in the activity of SOD and CAT at any of the ethanol concentrations (Figure 4).

## 4. Discussion

In this experimental study, we aimed to determine the optimal ethanol concentration to serve as a vehicle for administering resveratrol at a concentration of 10 mg/kg/day. We observed that ethanol concentrations of 7.5% and 10% as vehicles showed the greatest positive effects. Resveratrol has been extensively studied for its beneficial properties; however, its bioavailability is known to be low, limiting its antioxidant function [28, 29]. Recent studies have shown that various vehicles can enhance resveratrol bioavailability, with ethanol being among the most effective [30]. Robinson et al. [31] demonstrated that ethanol increases the solubility of resveratrol more than other substances.

Numerous formulations have been explored to overcome the poor oral bioavailability of resveratrol. Early reports, such as that by Belguendouz et al. [32], highlighted its limited solubility in water (< 0.05 mg/mL), which severely restricts its therapeutic potential. Subsequent strategies have included structural modifications and alternative delivery systems, including solid lipid nanoparticles [33], solid dispersions with magnesium dihydroxide [34], and enzymatically coated nanocarriers [35], all aimed at enhancing systemic absorption. Some studies have prioritized vehicle optimization, such as the use of ethanol–saline mixtures [22], propylene glycol–water combinations [36], or polyethylene glycol [31], demonstrating improved solubility and bioefficacy. However, while ethanol has consistently improved solubility [18], high concentrations can also pose physiological risks [37]. Our study adds to this body of work by using a dose-gradient approach to identify an optimal ethanol concentration that balances solubility and biological safety in a translationally relevant model.

The 2-month treatment period used in this study is consistent with experimental models mimicking chronic alcohol exposure and its long-term effects on oxidative stress and neuroinflammation in the brain. Prolonged exposure is necessary to observe sustained alterations in antioxidant enzyme activity and neuronal function [38]. This timeframe also allows for a more accurate representation of the chronic effects of both ethanol and antioxidant interventions such as resveratrol.

Oral ethanol is metabolized in the liver by alcohol dehydrogenase (ADH2) to generate acetaldehyde as the primary metabolite. This compound is widely associated with oxidative damage in a concentration-dependent manner; however, this molecule is rapidly metabolized into less toxic substances such as acetate [39]. In addition, the brain is capable of metabolizing ethanol through CAT, cytochrome P450, and ADH enzymes [40]. These mechanisms suggest that low doses of ethanol used as a vehicle may not produce oxidative injury. Interestingly, our group previously reported that low-dose ethanol administration, even in the absence of resveratrol, was associated with changes in oxidative stress markers in the hippocampus [19, 20]. This observation supports the idea that ethanol itself may influence redox homeostasis at subtoxic levels, reinforcing the importance of evaluating its independent contribution when used as a pharmaceutical vehicle. The progressive ethanol gradient used in this study allowed for the systematic exploration of potential dose–response interactions while remaining within a pharmacologically acceptable and translationally relevant range.

Oral administration of resveratrol led to a reduced production of nitric oxide in the hippocampus, when 7.5% and 10% ethanol concentrations were added as seen in Figure 1. The hippocampus is known to be closely connected to other brain regions [5]. Nitric oxide is a molecule involved in various physiological functions and is produced by different isoforms of nitric oxide synthase (NOS) [41]. However, when NO is overproduced, particularly in combination with the superoxide anion, it can form peroxynitrite through a diffusion-limited reaction. This leads to nitrosative stress, as peroxynitrite is capable of oxidizing cellular components such as DNA, proteins, and lipids. These damaging effects extend to brain cells and may contribute to a wide range of pathological conditions, from atherosclerosis to neurodegenerative disorders [42, 43].

The protective effect of resveratrol is largely attributed to its antioxidant activity. It acts as a scavenger of oxidizing molecules and serves as an electron donor to stabilize free radicals, including hydroxyl radicals, superoxide anions, and nitric oxide [44]. By reducing nitric oxide levels, resveratrol may also lower the formation of other harmful molecules, such as nitrites and peroxynitrite, which exhibit high cellular toxicity. These results are consistent with findings reported by Navarro-Cruz et al. [20]. In turn, this reduction may prevent the chain reaction of fatty acid degradation that leads to the formation of toxic and carcinogenic metabolites, such as MDA and 4-HDA [45]. These metabolites were analyzed in this study and showed a similar decrease at ethanol concentrations of 7.5% and 10%.

The hippocampus is particularly susceptible to redox imbalance and oxidative injury, making it a key target for understanding the neurological impact of ethanol exposure and potential antioxidant interventions [46]. Although our study did not aim to model alcohol use disorder (AUD), prior research on ethanol-induced redox dysregulation highlights the relevance of antioxidant systems in the brain. For instance, proteomic studies in animal models have shown that ethanol consumption alters the expression of glutathione-related enzymes, which are keys in redox homeostasis [47]. This supports the importance of studying antioxidants such as resveratrol in contexts of ethanol exposure. Similarly, brain aging is associated with increased oxidative stress, mitochondrial dysfunction, and chronic glial activation, all of which impair neuronal homeostasis. Although our model used adult rats, the vulnerability of the hippocampus to oxidative damage aligns with mechanisms implicated in age-related neurodegeneration [48]. These parallels reinforce the relevance of studying hippocampal oxidative stress responses, even in nonaged models.

According to Kapetanovic et al. [11], only approximately 20% of orally administered resveratrol is absorbed, while the remainder is excreted in feces and urine. After absorption, resveratrol is rapidly metabolized into glucuronide and sulfate conjugates, which may reduce its antioxidant capacity.

Ethanol at concentrations of 7.5% and 10% appeared to influence the effects of resveratrol, potentially enhancing its antioxidant activity. However, the current experiments do not provide conclusive evidence that ethanol increases resveratrol's bioavailability. This possibility could be further explored by analyzing lipid peroxidation levels. In our study, a significant reduction in lipid peroxidation markers (MDA and 4-HDA) was observed with resveratrol treatment. In contrast, ethanol alone at those concentrations increased the levels of these markers compared to control groups. This effect may occur because, even at relatively low concentrations, ethanol could accumulate in the brain and contribute to increasing NO production and reactive oxygen species, which in turn generate lipid peroxidation byproducts. It is important to note that the primary role of vehicles such as ethanol is to avoid interacting with the active compound and to prevent cellular damage. Ethanol is commonly used as a pharmaceutical vehicle in concentrations ranging from 5% to 17%.

The antioxidant activity of resveratrol is attributed to its phenolic structure, which enables electron donation to stabilize reactive molecules such as NO and superoxide [49]. Our data are comparable with that presented by Kasdallah-Grissa et al. [50], who reported that resveratrol co-administered with ethanol reduced MDA + 4-HDA levels nearly to those of the control group in rat brain tissue. 4-HDA is a highly reactive lipid peroxidation product that interacts with thiol-containing amino acids and promotes neuronal apoptosis [51, 52].

The protection generated by the antioxidant on the fatty acids of the hippocampus is likely due to the use of ethanol as a vehicle in 7.5% and 10%, since it could promote the solubility of resveratrol, rapidly passing from the intestinal lumen to the bloodstream in a greater proportion. This effect may explain the reduction of lipoperoxidation products in greater proportion at a concentration of 7.5% ethanol.

Among the different mechanisms used by resveratrol are its direct antioxidant action or promotion of the *de novo* synthesis of enzymes that acts as antioxidants due to their ability to eliminate free radicals, which is why we evaluated the enzymatic activity of SOD and CAT [53].

SOD is a first-line of defense antioxidant, whose function is to dismutate the superoxide anion into hydrogen peroxide [3] to minimize the toxic effects that this molecule can cause when reacting with others. Our results indicate that the antioxidant effects of resveratrol were not uniform across all ethanol concentrations. Specifically, a significant increase in SOD activity was observed at 2.5% ethanol when co-administered with resveratrol, while CAT activity was significantly improved at both 2.5% and 7.5% ethanol. These findings suggest that the protective effects of resveratrol on enzymatic antioxidant systems may depend on the concentration of ethanol used as a vehicle. At other concentrations, no statistically significant changes were detected, indicating a possible threshold or nonlinear response. This highlights the importance of carefully selecting the vehicle concentration when evaluating the biological activity of polyphenols such as resveratrol. In addition, it could be explained by resveratrol counteracting the oxidizing molecules, avoiding the formation of more reactive ones. In the case of the superoxide anion, it is stabilized when resveratrol donates an electron, converting it into an O_2_ singlet. This means that SOD activity does not decrease, since the results showed that nitrite levels and lipid peroxidation products decreased almost to the values of the control group, suggesting that SOD continues to act as a primary antioxidant under normal conditions.

The results obtained showed that neither one of the two enzymes increased their enzymatic activity consistently across all groups. Therefore, the antioxidant activity of resveratrol is solely due to its molecular capacity as a scavenger of oxidizing molecules. Several in vivo studies conducted under conditions of chronic oxidative stress have reported heterogeneous effects of resveratrol on enzymatic antioxidant defenses such as SOD and CAT, with outcomes influenced by dose, treatment duration, and disease context. For example, in nonylphenol-exposed rats, administration of resveratrol was sufficient to restore both SOD and CAT activity in multiple tissues, suggesting a strong modulatory effect in toxicant-induced oxidative injury [54]. In high-fat diet models of metabolic syndrome, resveratrol improved renal antioxidant enzyme levels, in part through activation of the Nrf2 pathway [55]. Meta-analyses in diabetic nephropathy models have also revealed dose- and time-dependent patterns: higher doses are more effective in increasing SOD and glutathione, while shorter durations tend to preferentially enhance CAT activity [56]. These observations underscore the context-sensitive nature of resveratrol's antioxidant effects and suggest that the absence of consistent enzymatic upregulation in our hippocampal model may reflect differences in baseline oxidative stress, tissue specificity, and the pharmacodynamics of resveratrol at the tested dose and duration.

In ethanol-only groups, no consistent changes in SOD or CAT activity were observed compared to controls, although low doses (2.5% and 5%) tended to show mild increases in oxidative stress markers without significant enzymatic compensation. In contrast, when resveratrol was co-administered, SOD activity significantly increased at 2.5% ethanol and CAT activity increased at 2.5% and 7.5% ethanol. At higher ethanol concentrations (10%), neither SOD nor CAT showed significant differences compared to the ethanol-only groups, suggesting that the protective effect of resveratrol on enzymatic activity may be limited to low ethanol concentrations. Overall, neither enzyme demonstrated a consistent increase across all conditions, indicating that the main antioxidant effect of resveratrol is likely due to its direct radical scavenging capacity rather than sustained modulation of enzymatic systems.

Future studies should investigate the pharmacokinetic interaction between ethanol and resveratrol, particularly regarding its absorption and distribution in brain tissue. Evaluating the long-term effects of resveratrol under chronic ethanol exposure, as well as its role in modulating oxidative and inflammatory pathways relevant to neurodegenerative conditions, would provide valuable insight.

The present study has some limitations that should be taken into consideration for future research. The first is that bioavailability measurements of resveratrol in its active form were not performed. The second limitation is that there is no 100% reliability that demonstrates that resveratrol in its active form acts directly in the hippocampus and that it is not a peripheral response that indirectly reduces oxidative stress in the brain. The third limitation is that our study did not perform molecular tests demonstrating the regulation of the hippocampal redox microenvironment. The use of 5-month-old rats ensured the inclusion of mature adult physiology, which is particularly relevant for modeling chronic exposure scenarios and evaluating neuroprotective strategies in a more translationally relevant context [57].

## 5. Conclusions

Ethanol promotes oxidative stress progressively at concentrations of 5%, 7.5%, and 10%. The 7.5% ethanol concentration is the most appropriate for use as a vehicle to orally administer 10 mg/kg/day of resveratrol, since a 2-month administration of resveratrol significantly reduced the production of both nitrites and lipid peroxidation products at concentrations of 7.5% and 10%. However, resveratrol does not affect the enzymatic activity of SOD and CAT when administered with the different ethanol concentrations.

## Data Availability

The datasets generated and/or analyzed during the current study are available from the corresponding author on reasonable request.
